# Supplementary figures and images for: Fascin limits Myosin activity within Drosophila border cells to control substrate stiffness and promote migration
Source: eLife. 2021 Oct 26;10:e69836. doi: 10.7554/eLife.69836 (PMC8547955; doi:10.7554/eLife.69836)

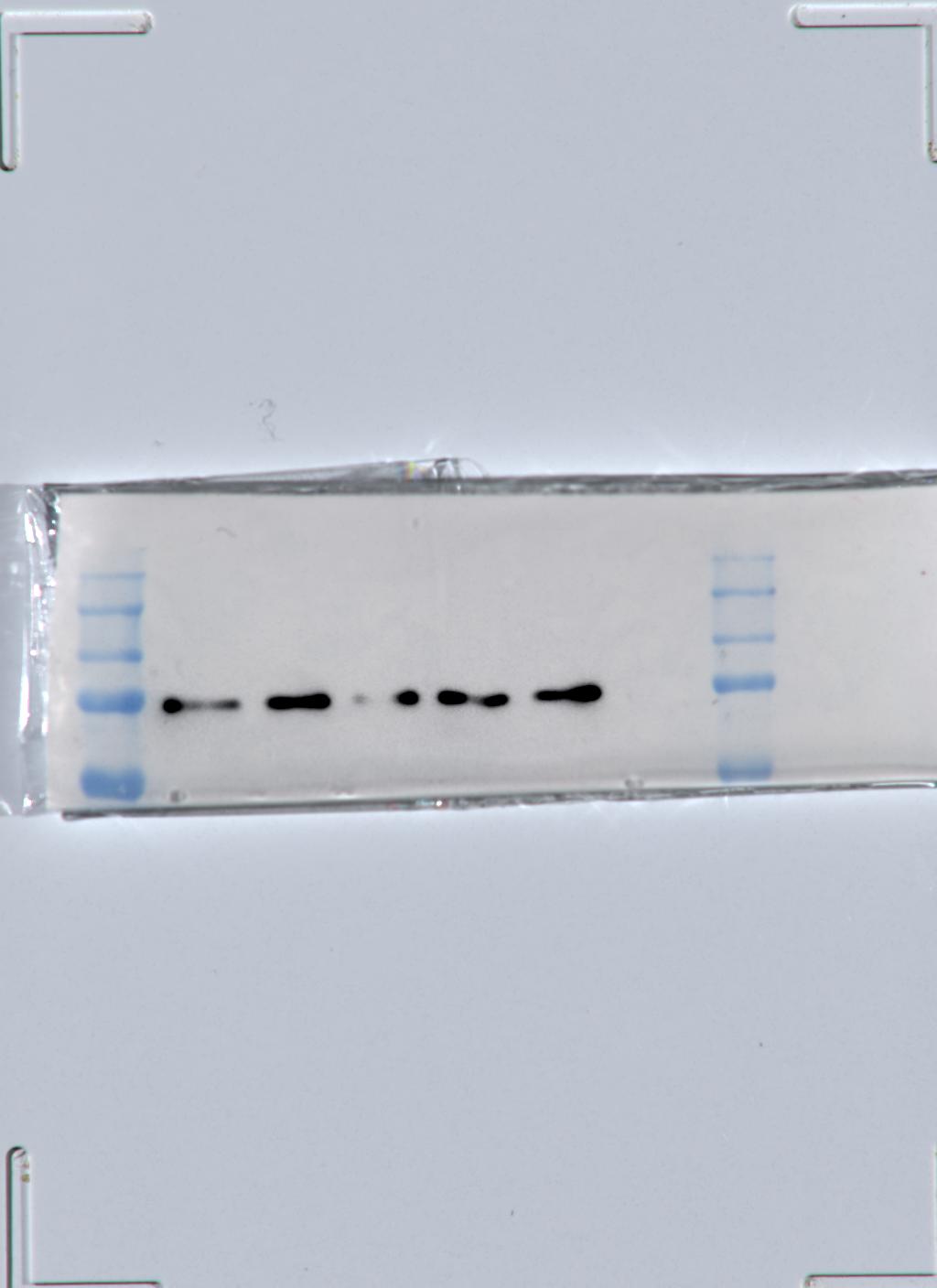

Supplement: Figure 2—figure supplement 1—source data 2. [file elife-69836-fig2-figsupp1-data2.zip › Figure 2- supplemental figure 1- source data/Figure 2 - supplemental figure 1 - source data 5.jpg]

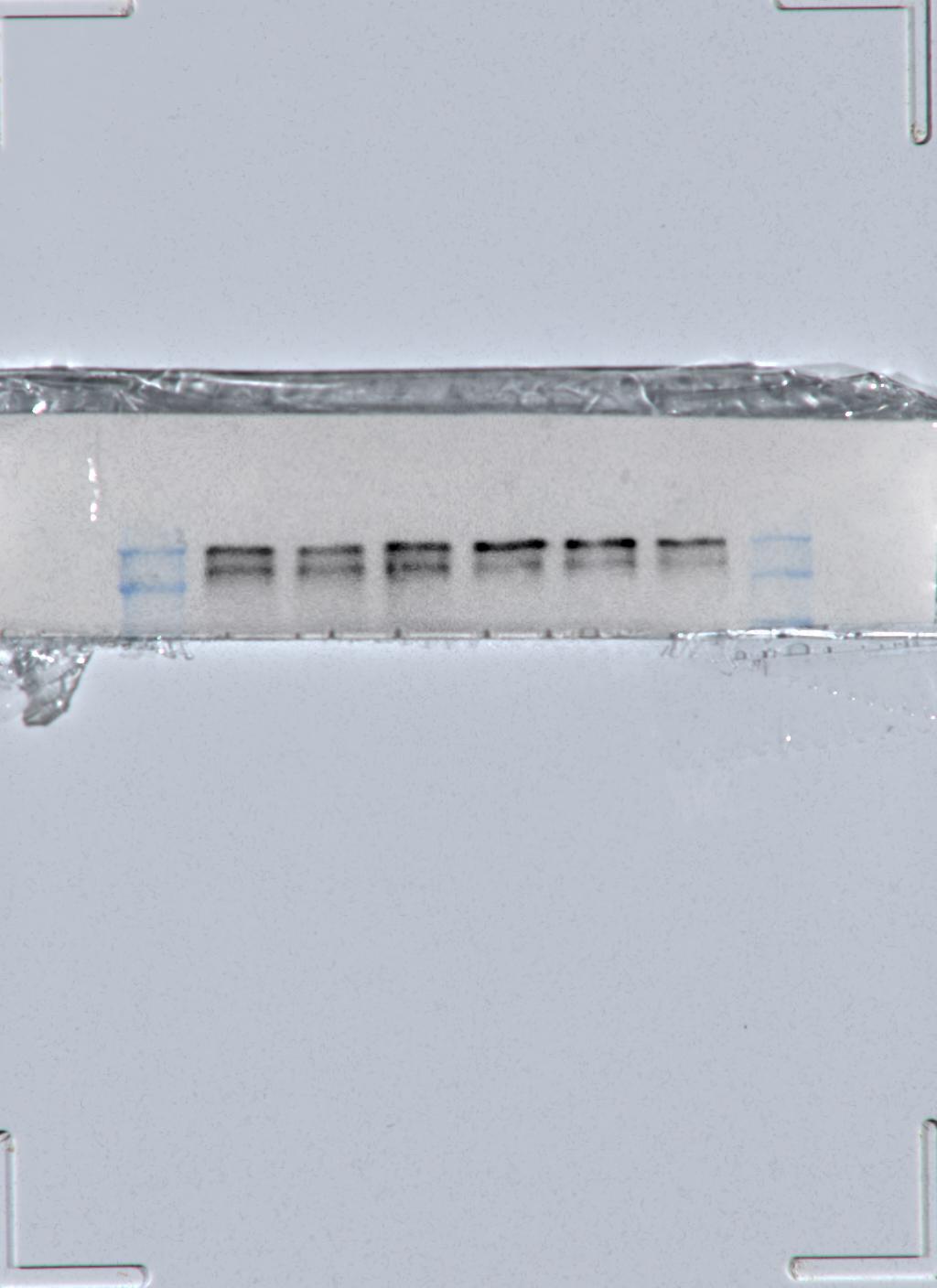

Supplement: Figure 2—figure supplement 1—source data 2. [file elife-69836-fig2-figsupp1-data2.zip › Figure 2- supplemental figure 1- source data/Figure 2 - supplemental figure 1 - source data 4.jpg]

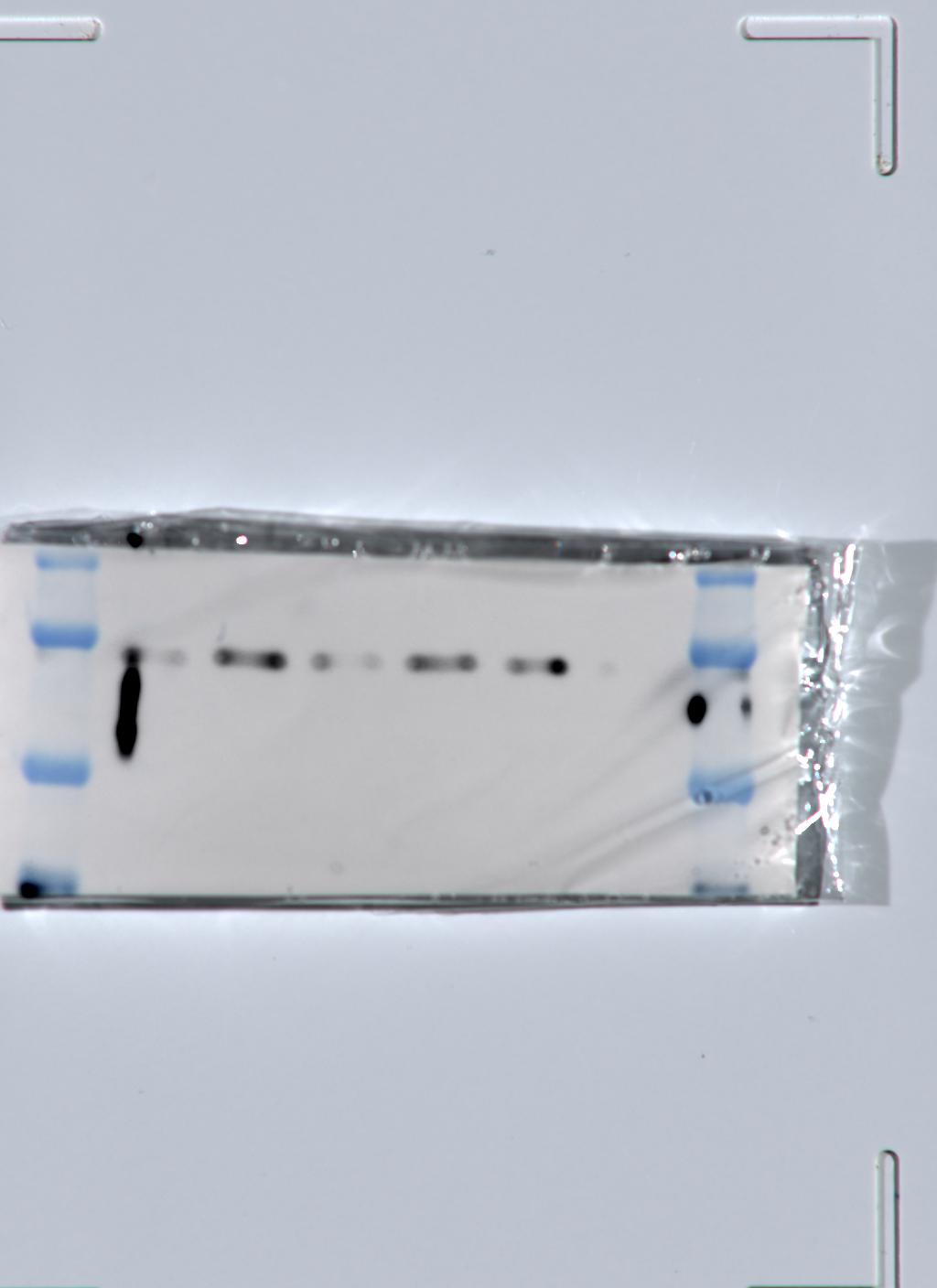

Supplement: Figure 2—figure supplement 1—source data 2. [file elife-69836-fig2-figsupp1-data2.zip › Figure 2- supplemental figure 1- source data/Figure 2 - supplemental figure 1 - source data 3.jpg]

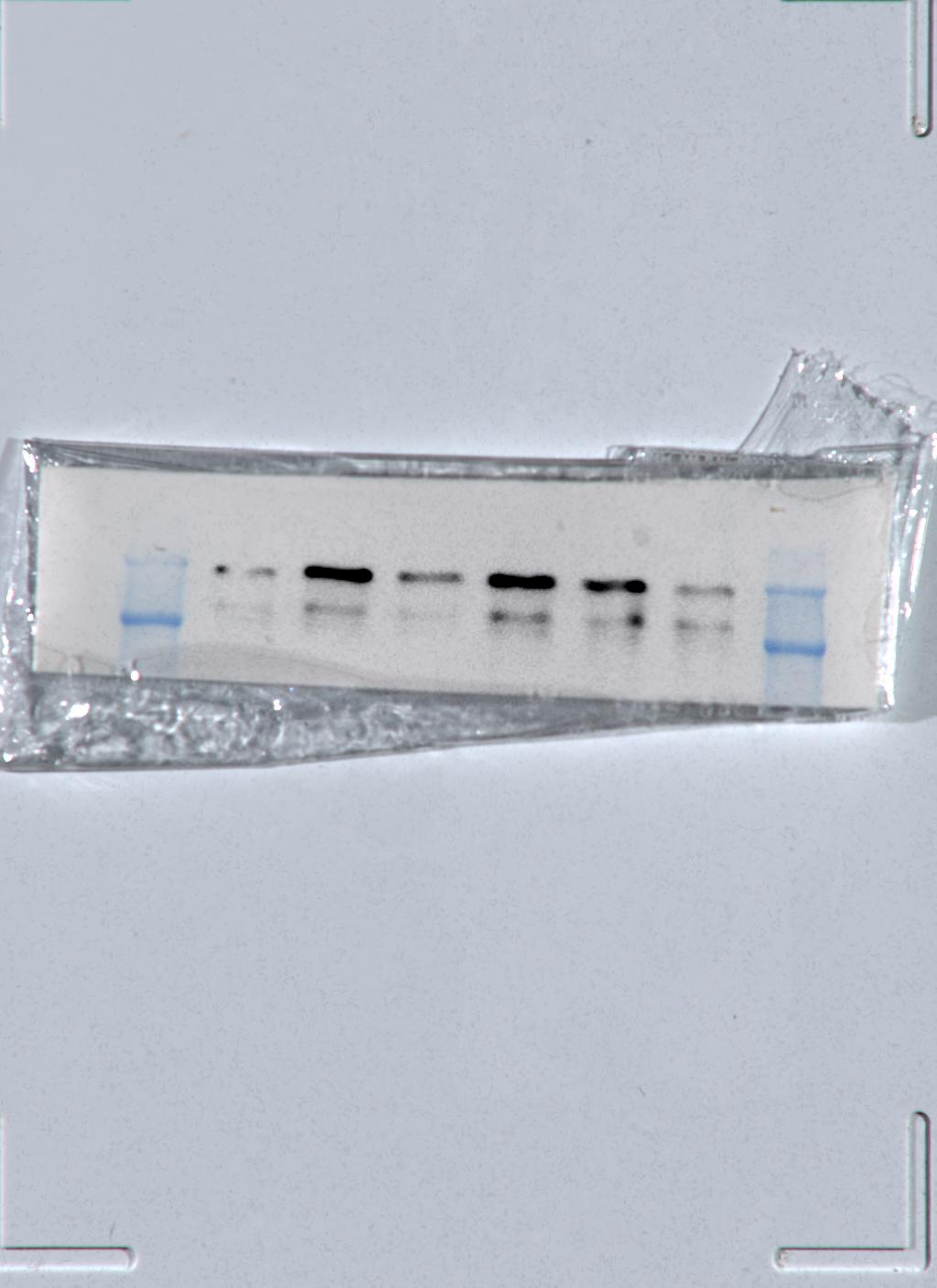

Supplement: Figure 2—figure supplement 1—source data 2. [file elife-69836-fig2-figsupp1-data2.zip › Figure 2- supplemental figure 1- source data/Figure 2 - supplemental figure 1 - source data 2.jpg]

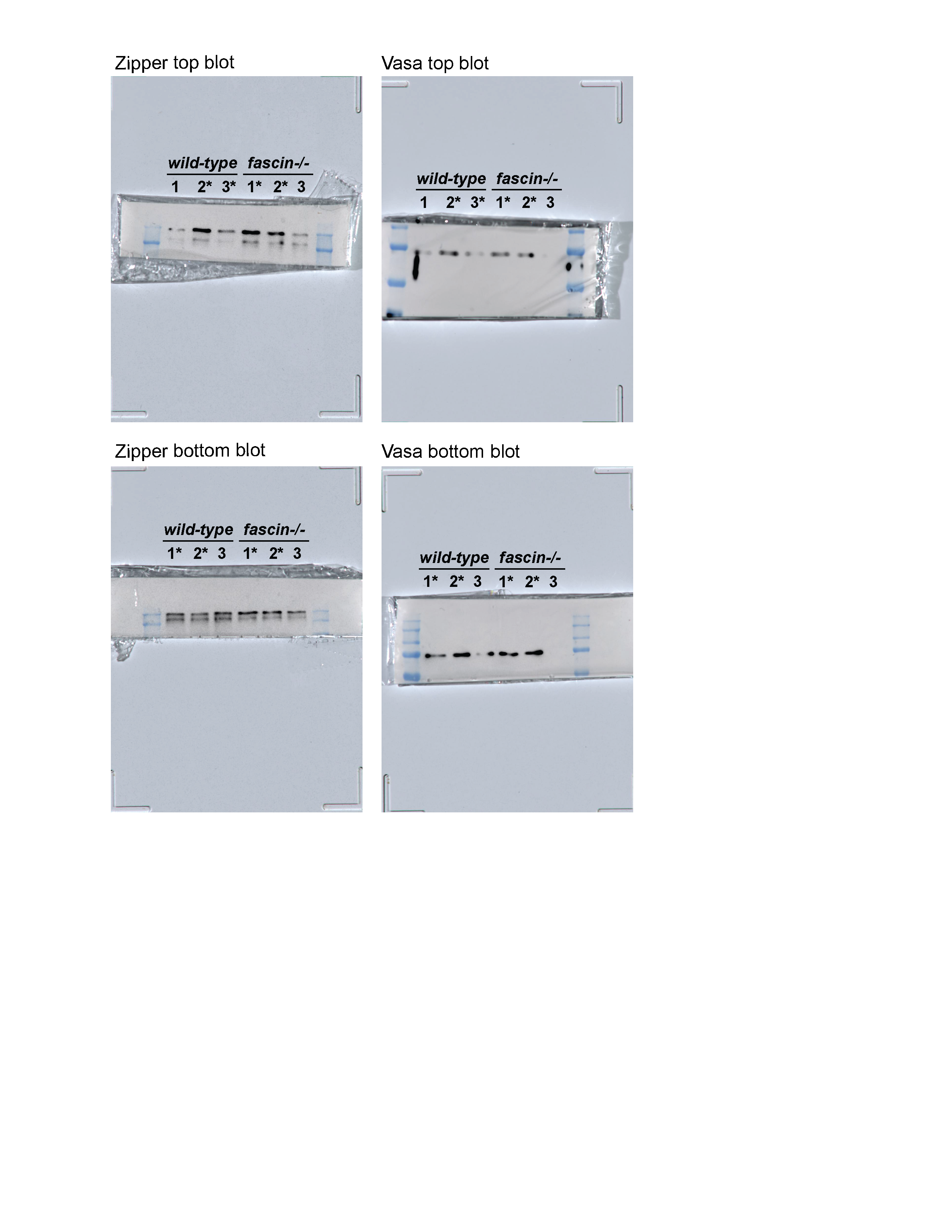

Supplement: Figure 2—figure supplement 1—source data 2. [file elife-69836-fig2-figsupp1-data2.zip › Figure 2- supplemental figure 1- source data/Figure 2 - supplemental figure 1 - source data 6.png]
